# NOVA1 acts as an oncogene in melanoma via regulating FOXO3a expression

**DOI:** 10.1111/jcmm.13527

**Published:** 2018-03-02

**Authors:** Xin Yu, Heyi Zheng, Matthew T.V. Chan, William K.K. Wu

**Affiliations:** ^1^ Department of Dermatology Peking Union Medical College Hospital Chinese Academy of Medical Sciences and Peking Union Medical College Beijing China; ^2^ Department of Anaesthesia and Intensive Care The Chinese University of Hong Kong Hong Kong City Hong Kong; ^3^ State Key Laboratory of Digestive Disease LKS Institute of Health Sciences The Chinese University of Hong Kong Hong Kong City Hong Kong

**Keywords:** FOXO3a, melanoma, NOVA1, RNA‐binding proteins

## Abstract

Increasing studies have suggested that dysregulation of RNA‐binding proteins (RBPs) contributes to cancer progression. Neuro‐oncological ventral antigen 1 (NOVA1) is a novel RBP and plays an important role in tumour development. However, the expression and role of NOVA1 in melanoma remain unknown. In this study, we indicated that NOVA1 expression was up‐regulated in melanoma samples and cell lines. Moreover, we demonstrated that knockdown of NOVA1 suppressed melanoma cell proliferation, migration and invasion in both A375 and A875 cell lines. In addition, we showed that suppressed expression of NOVA1 enhanced forkhead box O3a (FOXO3a) expression while inhibited AKT expression in melanoma cell. Furthermore, we demonstrated that inhibited expression of FoxO3A rescued NOVA1‐mediated cell proliferation, migration and invasion in melanoma cell line A375. These results suggested that NOVA1 acted as an oncogene in the development of melanoma partly through regulating FoxO3A expression.

## 1 INTRODUCTION

Melanoma is derived from melanocytes and responsible for most of skin tumour‐related deaths in human beings.^1^, ^2^, ^3^, ^4^, ^5^ There are about 76 380 new patients of melanoma and approximately 10 130 melanoma‐related deaths in 2016 in the United States.^6^ Although various treatments such as surgery, gene‐targeted therapy, chemotherapy, radiotherapy and immune therapy have been developed, the prognosis of cases with advanced melanoma is still unsatisfied.^2^, ^7^, ^8^, ^9^, ^10^ Unfortunately, the mechanism of the high rate of metastasis in melanoma remains unknown.^3^, ^11^, ^12^ Therefore, it is important to find new biomarkers and potential therapeutic targets for melanoma patients.

Recently, increasing studies have showed that RNA‐binding proteins (RBPs) are important for post‐transcriptional regulation.^13^, ^14^, ^15^, ^16^ Several studies have investigated the roles of RBPs in the prognosis and progression of melanoma.^17^, ^18^, ^19^ Neuro‐oncological ventral antigen 1 (NOVA1) is one member of RBPs and is involved in the programme of neural splicing.^20^, ^21^ The NOVA superfamily has two members such as NOVA1 and NOVA2. NOVA1 is expressed in central nervous system and can bind to the YCAY motif through its KH domain, thereby modulating alternative splicing.^22^, ^23^, ^24^ Growing evidence suggested that NOVA1 was deregulated in several tumours such as gastric cancer (GC), astrocytoma and oligodendroglioma, hepatocellular carcinoma (HCC) and lymphomas.^21^, ^25^, ^26^, ^27^ For example, Zhang et al^28^ demonstrated that overexpression of NOVA1 increased the HCC growth and retro‐regulated GABAARγ2 and GABA expression. Shen et al^29^ showed that ectopic expression of miR‐339 suppressed GC cell growth, invasion, migration and tumorigenicity through regulating NOVA1 expression. However, the expression and role of NOVA1 in melanoma remain unknown.

In this study, we firstly detected the expression of NOVA1 in melanoma tissues and control tissues. Then, we investigated the role of NOVA1 in melanoma cell. Furthermore, we explored the potential mechanism of NOVA1 in melanoma.

## 2 MATERIALS AND METHODS

### 2.1 Clinical specimens

Melanoma tissues and the normal adjacent samples were collected in our hospital. These samples were cut into two copies: One was fixed with the formalin for histopathological confirming, and the other was stored in liquid nitrogen. All patients provided written consent for this research experiment. Our study was approved by the Ethics Committee of Peking Union Medical College Hospital. The detailed clinical information was shown as follows: case 1: T3, ulceration, foot; case 2: T3, no ulceration, hand; case 3: T3, ulceration, foot.

### 2.2 Immunohistochemistry

Tissues were fixed with the 4% paraformaldehyde and then washed with ddH2O and PBS. Next, H2O2 (3%) was added and incubated at RT for a half‐hour and the tissue was washed with PBS for three times. Then, the tissue was incubated with 0.3% Triton X‐100 for 30 minutes, followed by incubation in 12% normal goat serum. The slides were incubated with anti‐NOVA1 primary antibodies (1:500, dilution, Abcam) and then incubated with biotin‐conjugated goat anti‐rabbit IgG. The slide was dehydrated and mounted, and figure was captured by microscopy.

### 2.3 Cell culture and cell transfection

Human melanoma cell lines (A375, A875 and SK‐MEL‐1) and normal melanocyte cell line (D78) were collected from Cell bank Center of Institute of Chinese Academy of Medical Sciences and Peking Union Medical College (http://www.cellresource.cn/cellsearch.aspx) (Beijing, China). These cell lines were cultured in the Dulbecco's modified Eagle's medium (DMEM) supplemented with FBS (foetal bovine serum) and penicillin and streptomycin and were kept at 37°C in the humidified atmosphere (5% CO2). Cells were transfected with the siRNA‐NOVA1 and siRNA‐control; siRNA‐forkhead box O3a (FOXO3a) and siRNA‐control plasmid using Lipofectamine 2000 (Invitrogen, CA, USA) according to the instruction.

### 2.4 RNA isolation and qRT‐PCR

Total RNA from cell or samples was extracted using Trizol reagent (Invitrogen, Carlsbad, CA, USA). cDNA was carried out by the M‐MLV reverse transcriptase (Invitrogen, CA, USA) from 1 ug total RNA, and qRT‐PCR was performed to determine the expression of NOVA1 and GAPDH using SYBR Green Kit (Qiagen) on the iQ5 Real‐Time PCR assat System (Bio‐Rad, USA) following the manufacturer's instructions. GAPDG was selected as the internal control. The mRNA expression was detected using 2^−DDCT^ method. The corresponding PCR primer was shown as follows: NOVA1, forward, 5′‐GGTCTCAGCCAAGCAGCAGCAA‐3′ and reverse, 5′‐TTGCAGCAGTAGCAGCAGCCAG‐3′; GAPDH, forward, 5′‐TGGAT CTGACATGCCGCCTGGA‐3′ and reverse, 5′‐AGGTC CACCA CCCTGTTGCTGT‐3′.

### 2.5 Western blotting

Protein lysates from cells or tissues were prepared using the RIPA kit (Beyotime, China) following the manufacturer's instruction. The protein concentration was measured by BCA protein kit. Protein was separated by 12% SDS‐PAGE and transferred to PVDF membrane. After blocking with non‐fat milk, the membrane was immunoblotted with primary antibodies (anti‐NOVA1, anti‐FoxO3A and anti‐AKT, diluted 1:1000) overnight. After washing for three times, the membrane was incubated with HRP‐linked secondary antibodies for 1 hour. The signal was measured by enhanced chemiluminescence (ECL) (Pierce, USA). GAPDH was used as the internal control.

### 2.6 Cell proliferation, migration and invasion assay

Cell growth was monitored using a Cell Counting Kit‐8 (CCK‐8, Dojindo, Japan) following the manufacturer's information. Cells (1 x 10^5^ cells) were cultured on the 96‐well plate, and cell proliferation was measured at 0, 1, 2 and 3 days. The number of cells was determined by measurement of OD at 450 nm using microplate reader (TECAN). Scratch wound‐healing assay was used to measure cell migration. When cells were grown to confluence on the 12‐well plate, scratch wound was created using 200 μL pipette tip. Cells were cultured in serum‐free medium; picture was taken at 0, 48 and 60 hours. For cell invasion assay, transwell was used. Cells were seeded on the top side of transwell chamber coated with Matrigel in serum‐free medium; medium with 10% serum was added in the lower chamber. The invaded cells on the lower membrane were washed and stained with crystal violet.

### 2.7 Statistical analysis

All data were shown as mean ± SD (standard deviation), and statistical assay was performed using the SPSS 19.0 (SPSS Inc., Chicago, IBM). Student's *t*‐test and one‐way ANOVA were used to calculate the significance between different groups. *P* < .05 was indicated as significant.

## 3 RESULTS

### 3.1 NOVA1 expression was up‐regulated in melanoma tissues and melanoma cell lines

**Figure 1:**
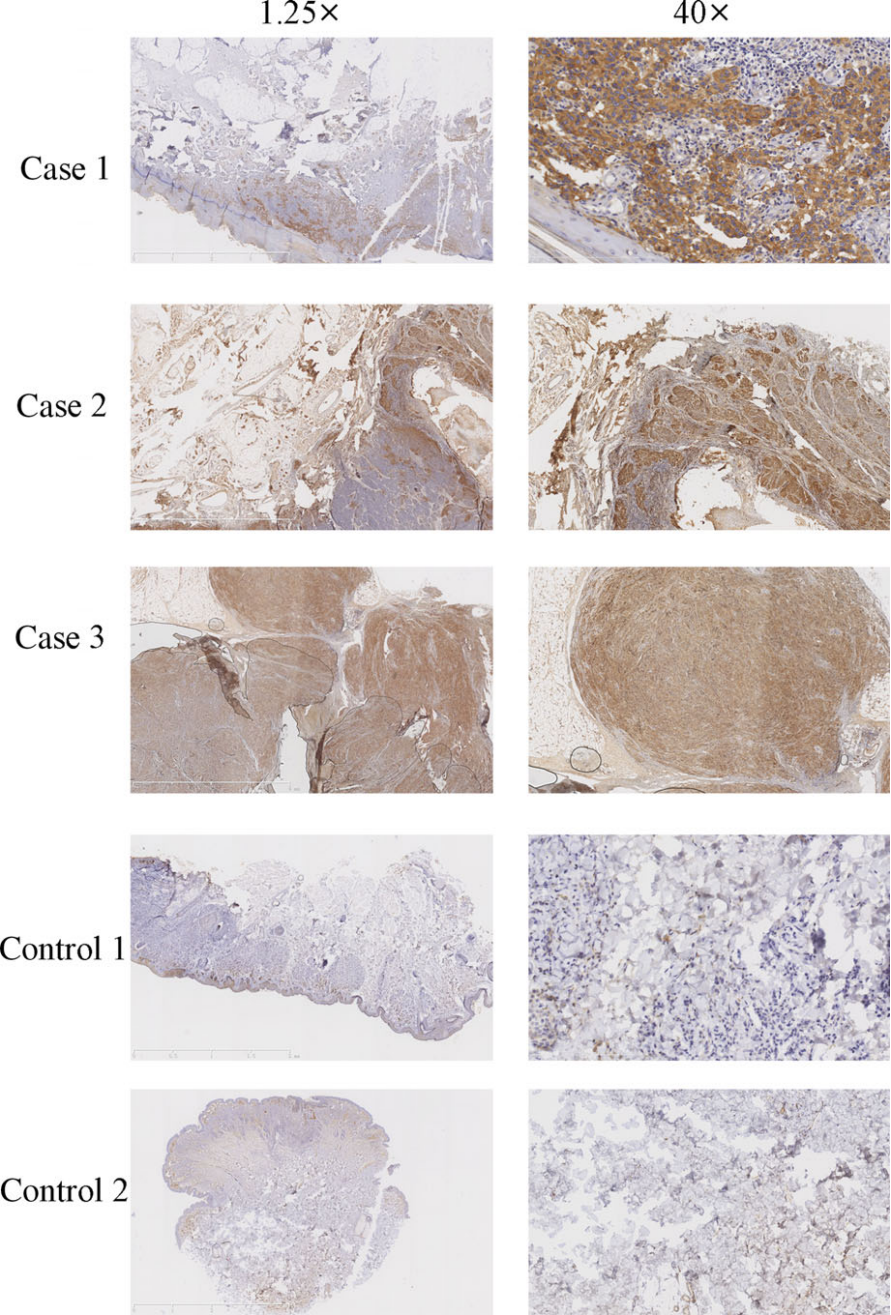
Neuro‐oncological ventral antigen 1 (NOVA1) expression was up‐regulated in the melanoma tissues. The expression of NOVA1 in the three patients with melanoma samples and two patients of cutaneous nevus was measured using immunostaining analysis. Magnification: 1.25× and 40×. The brown was indicated as the positive of NOVA1

**Figure 2:**
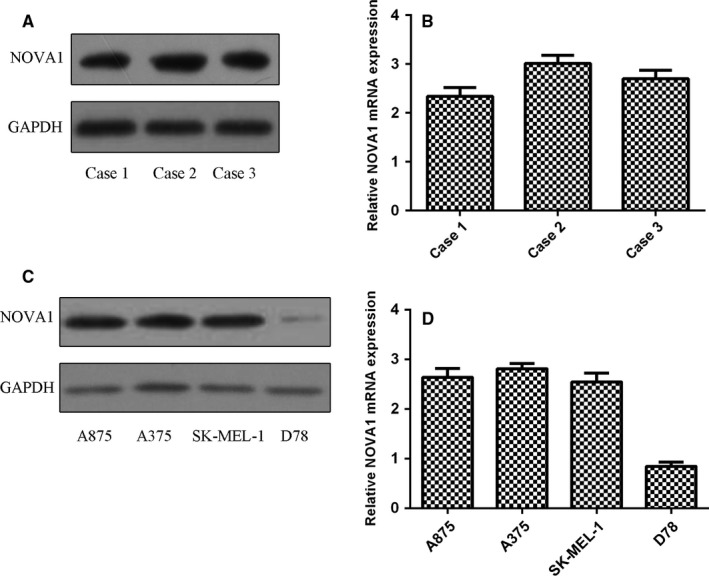
Neuro‐oncological ventral antigen 1 (NOVA1) expression was up‐regulated in the melanoma cell lines. A, The protein expression of NOVA1 in these three cases with melanoma tissues was determined by Western blot. B, The mRNA expression of NOVA1 in these three cases with melanoma tissues was determined by qRT‐PCR. C, The protein expression of NOVA1 in the melanoma cell lines (A875, A375 and SK‐MEL‐1) and normal melanocyte cell line (D78) using Western blot. D, The mRNA expression of NOVA1 was measured by qRT‐PCR

Firstly, we measured the expression of NOVA1 in three patients with melanoma and two cases of cutaneous nevus using immunostaining analysis. Our result showed that the expression of NOVA1 in melanoma was overexpressed in all three patients with melanoma compared to that in two cases of cutaneous nevus (Figure 1). Furthermore, we detected NOVA1 expression in melanoma tissues using qRT‐PCR and Western blot. Our data showed that NOVA1 protein was expressed in three melanoma samples (Figure 2A). In line with this, NOVA1 mRNA was also detected in three melanoma tissues (Figure 2B). In addition, we demonstrated that the expression of NOVA1 was up‐regulated in melanoma cell lines (A875, A375 and SK‐MEL‐1) compared to normal melanocyte cell line (D78) using Western blot (Figure 2C) and qRT‐PCR (Figure 2D).

### 3.2 Knockdown of NOVA1 suppressed melanoma cell proliferation

**Figure 3:**
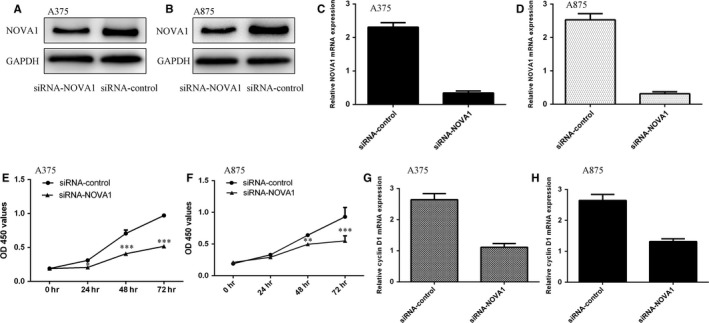
Knockdown expression of NOVA1 suppressed the melanoma cell proliferation. A, The protein expression of NOVA1 in the melanoma cell line A375 was determined by Western blot. B, The protein expression of NOVA1 in the melanoma cell line A875 was determined by Western blot. C, The mRNA expression of NOVA1 in the melanoma cell line A375 was determined by qRT‐PCR. D, The mRNA expression of NOVA1 in the melanoma cell line A875 was determined by qRT‐PCR. E, Inhibition expression of NOVA1 suppressed the A375 cell growth. F, Down‐regulation expression of NOVA1 inhibited A875 cell proliferation. G, The mRNA expression of cyclin D1 in the A375 was measured by qRT‐PCR. H, The mRNA expression of cyclin D1 in the A875 was measured by qRT‐PCR. **P < .01 and ***P < .001

To explore the functions of FoxO3a in melanoma progression, siRNA‐NOVA1 was transfected into melanoma cell lines (A375 and A875). As shown in Figure 3A,B, the expression of NOVA1 was significantly decreased in A375 and A875 cells after treated with siRNA‐NOVA1. In addition, the mRNA expression of NOVA1 was inhibited in A375 and A875 cells treated with siRNA‐NOVA1 (Figure 3C,D). Moreover, inhibited expression of NOVA1 suppressed A375 (Figure 3E) and A875 (Figure 3F) proliferation using the CCK‐8 assay. We also demonstrated that down‐regulation of NOVA1 suppressed the cyclin D1 expression in the A375 (Figure 3G) and A875 cells (Figure 3H).

### 3.3 Inhibited expression of NOVA1 suppressed melanoma cell migration and invasion

**Figure 4:**
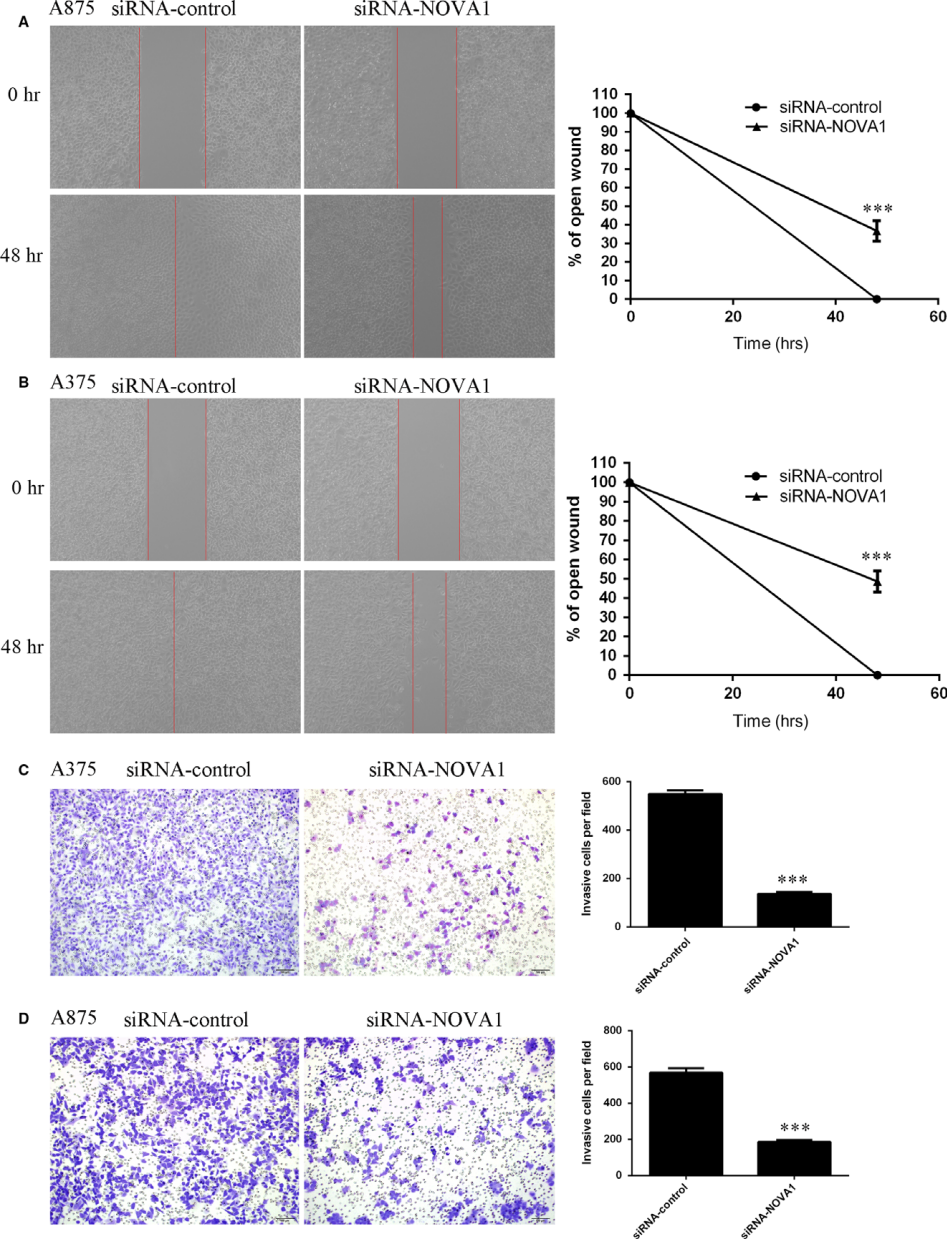
Inhibition expression of NOVA1 inhibited the melanoma cell migration and invasion. A, Scratch wound‐healing assay was performed to determine the cell migration. The relative ratio of wound closure was shown in the right. B, Inhibition expression of NOVA1 inhibited the A375 cell migration, and the relative ratio of wound closure was shown in the right. C, Down‐regulation expression of NOVA1 suppressed the A375 cell invasion using transwell assays. D, Inhibition expression of NOVA1 decreased the A875 cell invasion. ***P < .001

Furthermore, we demonstrated that suppressed expression of NOVA1 inhibited A875 cell migration using wound‐healing assay (Figure 4A), and the relative ratio of wound closure was shown in the right. In addition, we also showed that inhibited expression of NOVA1 suppressed A375 cell migration, and the relative ratio of wound closure was shown in the right (Figure 4B). Moreover, down‐regulated expression of NOVA1 suppressed the A375 cell invasion using transwell assays (Figure 4C). In line with this, we also showed that inhibited expression of NOVA1 decreased A875 cell invasion (Figure 4D).

### 3.4 NOVA1 involvement in FoxO3A and AKT expressions

**Figure 5:**
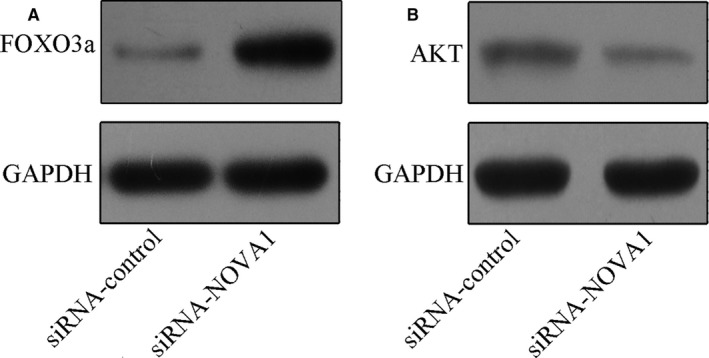
Knockdown expression of NOVA1 enhanced the FoxO3A expression and decreased AKT expression. A, The protein expression of FoxO3A was determined in the A375 cell by Western blot. B, The protein expression of AKT was determined in the A375 cell by Western blot

Next, we studied the mechanism of NOVA1 in melanoma. We showed that knockdown of NOVA1 enhanced FoxO3A expression in melanoma A375 cell (Figure 5A). Moreover, we indicated that down‐regulated expression of FoxO3A inhibited the AKT expression in A375 cell (Figure 5B).

### 3.5 Inhibited expression of FoxO3A rescued NOVA1‐mediated cell proliferation, migration and invasion

**Figure 6:**
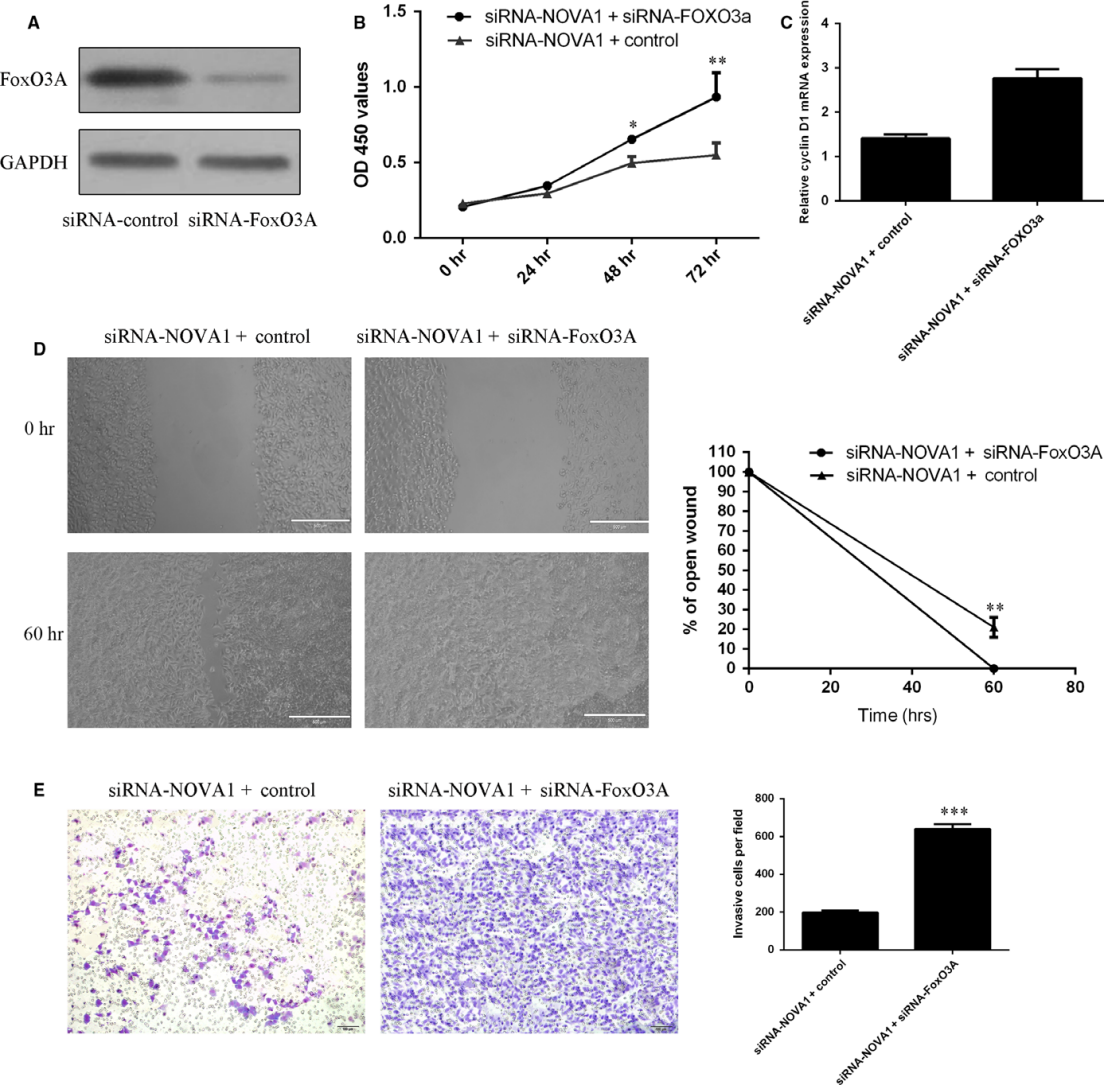
Inhibition expression of FoxO3A rescued NOVA1‐mediated cell proliferation, migration and invasion. A, The protein expression of FoxO3A was determined in the A375 cell by Western blot. B, Cell proliferation was determined using CCK‐8 analysis. C, The mRNA expression of cyclin D1 was measured by qRT‐PCR. D, Inhibition expression of FoxO3A partly rescued NOVA1‐mediated cell migration. E, Cell invasion was determined in the A375 cell using transwell assay. *P < .05, **P < .01 and ***P < .001

To study the role of FoxO3A in NOVA1‐regulated melanoma cell proliferation, migration and invasion, A375 was treated with siRNA‐FoxO3A. We confirmed that the expression of FoxO3A was significantly down‐regulated in the A375 cell after treated with siRNA‐FoxO3A (Figure 6A). Moreover, we showed that inhibited expression of FoxO3A partly rescued NOVA1‐mediated cell proliferation (Figure 6B). In line with this, we showed that suppressed expression of FoxO3A promoted the cyclin D1 expression in the siRNA‐NOVA1‐treated A375 cell (Figure 6C). Furthermore, inhibited expression of FoxO3A partly rescued NOVA1‐mediated cell migration (Figure 6D). We also indicated that suppressed expression of FoxO3A enhanced cell invasion in the siRNA‐NOVA1‐treated A375 cell (Figure 6E).

## 4 DISCUSSION

We investigated the expression and functional role of NOVA1 in melanoma. Firstly, we indicated that NOVA1 expression was up‐regulated in melanoma samples and cell lines. Moreover, we demonstrated that knockdown of NOVA1 suppressed melanoma cell proliferation, migration and invasion in both A375 and A875 cells. In addition, we showed that suppressed expression of NOVA1 enhanced FoxO3A expression and suppressed AKT expression in melanoma cell line A375. Furthermore, we demonstrated that inhibited expression of FoxO3A rescued NOVA1‐mediated cell proliferation, migration and invasion in melanoma cell line A375. These results suggested that NOVA1 acted as an oncogene in the development of melanoma partly through regulating FoxO3A expression.

Growing evidence has proved that RBPs play crucial roles in cell biology such as cell growth, apoptosis, migration, differentiation and invasion.^30^, ^31^, ^32^ Several studies suggested that RBPs acted crucial roles in the development of many tumours.^32^, ^33^ NOVA1 is one neuron‐specific RBP, which can influence the ligand‐binding, electrophysiological and signal transducing properties.^20^, ^34^, ^35^ Recently, several references have demonstrated that NOVA1 plays an important role in the progression of cancers. For example, Zhang et al^36^ indicated that NOVA1 enhanced hepatocellular carcinoma growth in vivo partly because of its interaction with the GABAA Receptor‐γ2. Kim et al^25^ demonstrated that suppressed expression of NOVA1 was found in the gastric cancer (GC) microenvironment, and down‐regulated expression of NOVA1 in GC cells was correlated with GC progression and poor prognosis. Yoon et al^37^ showed that NOVA1 was one candidate biomarker for prognosis of GC, which was regulated by miR‐146b‐5p. Zhi et al^26^ demonstrated that miR‐181b‐5p suppressed the astrocytoma growth, invasion and migration and enhanced cell apoptosis partly through inhibiting NOVA1 expression. However, the expression and functional role of NOVA1 in melanoma are still uncovered. Therefore, we firstly detected the expression of NOVA1 in melanoma samples and cell lines. We found that NOVA1 expression was up‐regulated in melanoma tissues and cell lines. In addition, we indicated that inhibited expression of NOVA1 suppressed melanoma cell growth, migration and invasion.

Previous study showed that suppressed expression of NOVA1 enhanced FoxO3A expression and inhibited AKT expression in the pancreatic beta cell.^38^ As a crucial transcription factor, FOXO3a was a downstream factor which was negatively modulated by PI3K/AKT signal pathway in several human tumours and the p‐FOXO3a catalysed by AKT phosphorylation will significantly inhibit FOXO3a transcriptional activity.^39^, ^40^, ^41^ FoxO3a was shown as a transcription factor which was involved in modulation of the apoptosis, stress response and autophagy.^42^, ^43^ Recently, a study showed that inhibited expression of FOXO3a enhanced tumour metastasis and was negatively correlated with the metastasis‐free survival in cases with clear cell renal cell carcinoma.^44^ Yan et al^45^ demonstrated that ectopic expression of FoxO3a attenuated basal migration and invasion of uveal melanoma. In our study, we demonstrated that knockdown expression of NOVA1 enhanced the FoxO3A expression and decreased AKT expression. In addition, we showed that inhibited expression of FoxO3A rescued NOVA1‐mediated cell proliferation, migration and invasion. These results suggested that NOVA1 enhanced the melanoma cell growth, migration and invasion partly through suppressing the FoxO3A expression.

In conclusion, we indicated that NOVA1 expression was up‐regulated in melanoma tissues and cell lines. Moreover, we demonstrated that knockdown of NOVA1 suppressed melanoma cell proliferation, migration and invasion partly through regulating FoxO3A expression. NOVA1 acts an oncological role in melanoma, and knockdown may provide a novel therapeutic target for melanoma.

## CONFLICT OF INTEREST

The authors declare no competing financial interests.
